# Uncommon Presentation of Osteochondroma in a Flat Bone of Left Iliac Blade Lesion: A Case Report

**DOI:** 10.31729/jnma.8949

**Published:** 2025-04-30

**Authors:** Sushant Shah, Sujan Sharma, Bishal Panthi, Govinda Bhandari, Aron Shrestha, Lalita Poudel Chettri, Abhishek Kumar Thakur

**Affiliations:** 1 Maharjgunj Medical Campus, Maharjgunj, Kathmandu, Nepal; 2 Department of Pathology, Maharjgunj Medical Campus, Maharjgunj, Kathmandu, Nepal; 3 Department of Orthopaedics and Trauma Surgery, Maharjgunj Medical Campus, Maharjgunj, Kathmandu, Nepal

**Keywords:** *osteochondroma*, *iliac osteochondroma*, *pelvic osteochondroma*, *benign bone tumour*

## Abstract

Osteochondromas are the most common benign bone tumors, frequently affecting the metaphysis of long bones. Their occurrence in flat bones is rare, accounting for only 5% of cases. This report presents a solitary osteochondroma of the iliac blade, a rare anatomical location. A 17-year-old male presented with a progressively enlarging, painless bony mass on the hip, associated with lower back pain. Imaging studies, including ultrasound and MRI, revealed a bony outgrowth with medullary and cortical continuity and a benign cartilage cap. The patient underwent en bloc excision biopsy under spinal anesthesia, removing the lesion along with a portion of the iliac crest. Osteochondromas in flat bones are rare, posing diagnostic challenges. While imaging provides critical diagnostic clues, histopathology remains essential for confirmation. Surgical excision is the treatment of choice for symptomatic cases. This case highlights the importance of recognizing osteochondromas in unusual locations and employing multidisciplinary approach for optimal diagnosis and management.

## INTRODUCTION

Osteochondromas represent 20-50% of benign bone tumors and 9% of all bone tumors, making them the most prevalent type of bone tumor.^1^ They originate from the bone's outer layer and are developmental abnormalities rather than actual tumors.^2^ Osteochondroma is a solid, immovable mass that typically arises near the end of a long bone at the metaphysis of the distal femur, proximal radius, proximal tibia, and proximal fibula.^3^ Genetic transmission is autosomal dominant and can cause solitary lesions or many growths.^4^ Reid originally reported solitary osteochondroma (also known as osteocartilaginous exostosis or exostosis) and it makes up 10% of all primary osseous tumors.^5,6^ Pelvic osteochondromas are rare, representing 5% of cases.^1^ Surgical excision is indicated for cosmetic concerns, pressure effects, or malignant transformation into chondrosarcoma.^7^

In this case report, we present a solitary osteochondroma in an adult located on the left iliac blade with associated low back pain.

## CASE REPORT

A 17-year-old male presented to our tertiary care center with a complaint of a progressively enlarging mass on his left hip, which he had first noticed over a year ago. The mass was initially detected incidentally during an MRI performed for Guillain-Barre syndrome evaluation. Over time, the patient observed a gradual increase in its size. On examination, a hard, non-tender mass was palpable in the left loin region. Although the patient reported a recent onset of lower back pain exacerbated by walking and jolting movements, the range of motion in the right hip remained fully preserved. There was no history of similar complaints in the family or any associated comorbidities.

**Figure 1:**
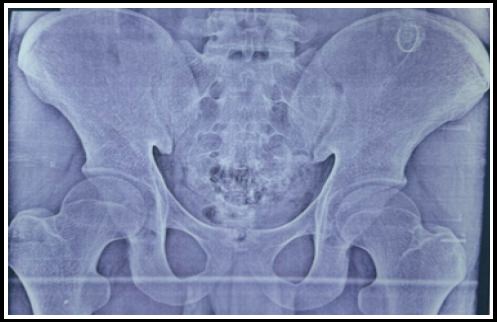
X ray showing osseous mass on left iliac blade.

X-ray imaging revealed an osseous mass located on the left iliac blade. An ultrasound of the left lower back demonstrated a bony structure measuring 2.2 × 2.1 cm overlying the left iliac crest, aiding in further localization and assessment. To further characterize the lesion, an MRI of the pelvis was performed, confirming a well-defined bony outgrowth measuring approximately 2.1 × 1.6 cm with medullary and cortical continuity with the parent bone. The MRI also revealed a cartilaginous cap thickness of 5 mm, which is within the benign range. Based on these imaging findings, a diagnosis of osteochondroma of the flat bone was established, and a management plan was formulated accordingly (Figure 1).

**Figure 2:**
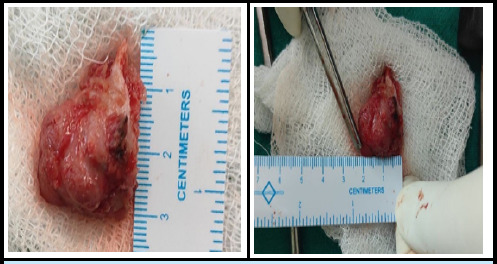
Excised mass from left iliac blade of 3*2cm.

After completing the necessary pre-operative examinations, obtaining pre-anesthetic fitness clearance, and securing informed consent, the patient was scheduled for an en bloc excision biopsy of the tumor. Under spinal anesthesia, the patient was positioned in a proper lateral posture. Following aseptic precautions, the surgical site was painted and draped. A 6-7 cm incision was made over the palpable mass. Upon dissection of the soft tissues, a bony mass measuring approximately 2 × 3 cm was identified on the posterior superior aspect of the left iliac blade. The pedicle and base of the mass were carefully explored, and the entire lesion, including part of the iliac crest, was excised. The resected mass was sent for histopathological examination, and the incision was closed with sutures. The actual size of the osteochondroma was documented as 3 × 2 cm (Figure 2).

**Figure 3:**
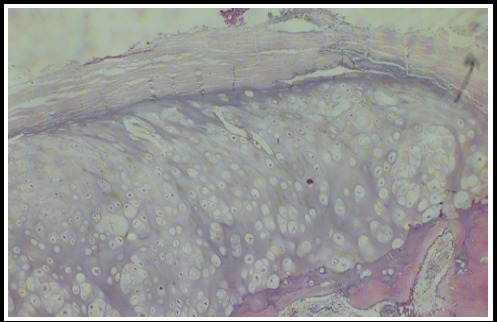
100 X histopathology showing encapsulated mass of mature hyaline cartilage and bony trabeculae underlying it.

**Figure 4:**
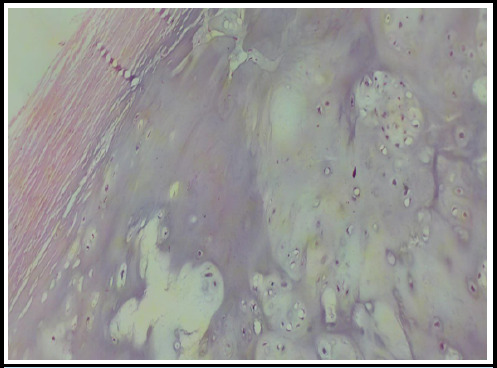
200 X histopathology showed fibrous perichondrium and hyaline cartilage.

Microscopic examination of the biopsy specimen revealed a cap composed of mature hyaline cartilage covered by a fibrous perichondrium. The cartilaginous cap demonstrated endochondral ossification into bone, with intervening marrow spaces between the bony trabeculae containing hematopoietic elements (Figure 3 and 4). No atypical cells were identified. Based on the histopathological findings, which serve as the gold standard for diagnosis, the patient was confirmed to have osteochondroma of the left iliac blade.

The patient showed excellent recovery following the surgical excision of the osteochondroma. At the 6-week follow-up, he was pain-free, with the surgical wound fully healed and no recurrence of symptoms. He resumed daily activities, including walking, without difficulty, and hip mobility was preserved.

At the 3-month follow-up, the patient remained symptom-free and fully functional, with no signs of recurrence or complications. Long-term prognosis is favorable due to the complete excision of the lesion. Annual follow-ups were recommended to monitor for any late-onset issues, though the risk of recurrence is minimal.

## DISCUSSION

Osteochondromas, the most common benign bone tumors, typically arise from the metaphysis of long bones primarily distal femur, proximal radius, proximal tibia, and proximal fibula.^8,9^ However, their occurrence in the pelvis, scapula, skull, hand and feet is relatively uncommon.^8,10^ This case report highlights the rare clinical presentation of pelvic blade osteochondroma in a 17 yrs old boy with diagnostic challenges.

The patient presented with a long-standing mass on the left hip, which had been gradually increasing in size over more than 1 year. This slow growth pattern is characteristic of osteochondromas.^11^ The absence of tenderness and the preservation of hip motion are consistent with the benign nature of the tumor. The patient's recent onset of lower back pain, aggravated by walking, suggests a mass effect, which is a common complication when osteochondromas grow large enough to impinge on surrounding structures.^10^ Osteochondromas can cause symptoms by compressing nearby structures, leading to nerve-related numbness, vascular issues like thrombosis or aneurysms, and changes in limb color and the popliteal artery, common peroneal nerve, and posterior tibial nerve are most often impacted.^12^ These symptoms are mostly associated with large size and long standing which were absent in our case.

Imaging studies played a crucial role in the diagnosis. Ultrasound initially identified an osseous structure overlying the left iliac crest. Revision MRI further characterized the lesion, revealing a well-defined bony outgrowth with medullary and cortical continuity with the parent bone, and a cartilage cap, which are hallmark features of osteochondroma.^13,14^ The size and characteristics of the cartilage cap are important, a cap of less than 3 cm suggests benign lesion, as a cap thicker than 3 cm in may suggest malignant transformation.^13^ In this case, the cartilage cap measured approximately 3.0 mm, indicating a benign lesion and chondrosarcoma was ruled out. In our case, we found the pedicle connecting the tumor to the iliac crest on CT, and there was no evidence of bone destruction and invasion of the surrounding tissue suggesting of no malignant changes.^14^ Biopsy is the gold standard for the diagnosis.

Conservative treatment options for osteochondromas include immobilization with splints, physiotherapy, NSAIDs, and local anesthetic injections. However, when these methods are ineffective, surgical removal of the tumor under anesthesia becomes necessary. Surgical excision is the treatment of choice for symptomatic osteochondromas as in our case or those causing complications like pain, restriction in movement, neurovascular compression and cosmetic problem.^10,14^ In this case, en bloc excision biopsy successfully removed the lesion along with part of the iliac crest, and histopathology confirmed benign osteochondroma.

The prognosis for osteochondroma is excellent, with a <2% recurrence rate following complete excision and a ~1% risk of malignant transformation in solitary cases. Post-surgical complications occur in ~11.6— 12.5% of cases, with peroneal neurapraxia being the most common, along with arterial laceration, compartment syndrome, and fibula fracture. However, the overall surgical risk remains low and comparable to other elective procedures.^1^ The meticulous surgical approach in this case ensured complete lesion removal, minimizing the risk of recurrence and promoting optimal recovery.

## CONCLUSION

This case presents the importance of considering osteochondroma in the differential diagnosis of pelvic masses, despite its rare occurrence in this anatomical location. Careful clinical evaluation, combined with appropriate imaging modalities and a tailored surgical approach, are crucial for the successful management of these uncommon lesions.
